# The Challenges in Dementia Three-Level Prevention Services Through Integrated Care Theory: Insights from an Ethnographic Study

**DOI:** 10.5334/ijic.9106

**Published:** 2026-08-10

**Authors:** Taomei Zhang, Yueju Wang, Lili Li, Liping Tan, Lanshu Zhou, Huiling Li, Fengmei Tian

**Affiliations:** 1 Department of Nursing, The Second Affiliated Hospital of Soochow University, Suzhou, China; 2 School of Nursing, Medical College of Soochow University, Suzhou, China; 3 Department of Geriatrics, The First Affiliated Hospital of Soochow University, Suzhou, China; 4 Liu Yuan Community Hospital, Suzhou, Jiangsu, China; 5 School of Nursing, Naval Military Medical University, Shanghai, China

**Keywords:** dementia, integrated care, Rainbow model, ethnographic study, three-level prevention, communities

## Abstract

**Background:**

The number of older adults living with dementia is increasing in China and worldwide. Three-level prevention is vital to delay the occurrence and development of dementia. However, the current state of these services remains suboptimal. This study aimed to explore the programs and challenges in providing dementia prevention services.

**Methods:**

A one-year ethnographic study, including semi-participatory observations and in-depth interviews, was conducted in nine settings in China from July 2022 to June 2023. Twenty-four service providers and 26 residents or family members were observed, and seventeen of these 50 participants were interviewed. Data collection was guided by the Rainbow Model. A combination of deductive and inductive content analysis was applied to identify the challenges.

**Results:**

Dementia-friendly communities, Dementia Screening Program, Medical Consortia, and Long-term care services were the main programs in dementia three-level prevention services. This study identified 32 challenges, including four at system level, ten at organizational level, seven each at professional and clinical levels, and four additional challenges.

**Conclusions:**

Enriching the diversity of programs and strengthening primary prevention services will be beneficial for improving the dementia three-level services. The challenges identified in this study can provide valuable insights to guide targeted interventions, inform policy, and optimize service delivery for relevant stakeholders.

## Introduction

Dementia is a significant global public health concern. Worldwide, the number of people with dementia is projected to reach 78 million by 2030 and 140 million by 2050 [1]. In China, a study on dementia prevalence among adults aged ≥60 years estimated that approximately 15.07 million older adults are living with this condition [2]. The rising number of cases in China and globally highlights the increasing burden of this disease [1].

There is currently no cure for dementia, making a three-level prevention model the primary strategy to mitigate its occurrence and progression. This framework includes the following levels. Primary prevention, also known as etiological prevention, aims to eliminate or mitigate various risk factors to prevent the occurrence of dementia. Secondary prevention focuses on the early detection, diagnosis, and treatment of dementia to prevent or slow its progression. Tertiary prevention involves the clinical management and daily care for individuals with dementia, with the goal of providing systematic treatment and care guidance to improve their quality of life [3]. Related three-level prevention services include health education, screening, diagnosis, treatment, care support, etc [3]. However, the overall provision and management of these services remain inadequate. Modifying risk factors can reduce the incidence of cognitive impairment by up to 40% [3,4]. However, in practice, residents (hereafter referring to older adults in communities) have limited understanding of these risk factors and the early symptoms of cognitive impairment [5]. Consequently, most residents only seek specialized care after obvious symptoms, such as getting lost [6]. By this stage, their cognitive function has typically deteriorated to a moderate or severe level, and they have missed the optimal window for intervention. Once individuals are diagnosed with dementia, they often require various services such as medical, care, and information support services due to the complexity of their condition [7]. However, family support services following a diagnosis are often lacking [8]. Research indicates that residents with dementia and their families report that these services are often provided from different settings, resulting in fragmented care experience [9]. This lack of service continuity and coordination leads to low satisfaction among older adults with dementia, their caregivers, and even service providers [10]. Overall, the management and provision of three-level prevention services are often incomprehensive and uncoordinated for residents, and fragmented for those living with dementia and their families. Identifying the challenges in existing dementia three-level prevention services is crucial to optimizing care.

Integrated care, as a service design principle, has demonstrated significant potential to enhance care quality, alleviate care burden, and improve care experience. It addresses healthcare fragmentation by fostering collaboration among professionals and institutions, thereby improving the continuity of care [11,12]. For years, integrated care has been a widely discussed topic in healthcare management and health system design. This study adopts the World Health Organization’s definition of integrated care: “the management and delivery of health services such that people receive a continuum of health promotion, disease prevention, diagnosis, treatment, disease-management, rehabilitation and palliative care services, through the different levels and sites of care within the health system according to their needs throughout the life course” [13]. Specifically, we define integrated care as the means of ensuring that community-dwelling residents can access the dementia three-level prevention services across different levels and sites of the health and social care systems. The Rainbow Model of Integrated Care (RMIC), developed by Valentijn et al., provides a comprehensive framework for understanding the complexity of integrated care [14]. By placing people-focused and population-based care as the guiding principle of integration, the model encompasses integration processes at the macro (system integration), meso (organizational and professional integration), and micro (clinical integration) levels. Functional integration works alongside normative integration to ensure effective connectivity between the functioning of the integrated care system between various levels [14]. Therefore, it was chosen as the original framework for identifying the challenges of integrated care [15].

Addressing dementia as a public health priority, China released the National Action Plan (2024–2030) [16], which aims to establish a comprehensive and continuous system for dementia—encompassing prevention, screening, diagnosis, treatment, rehabilitation, and care for older adults—by 2030. Consequently, it is critical to examine the primary programs involved in the dementia three-level prevention services and the challenges associated with delivering comprehensive, accessible, continuous, and coordinated prevention services to residents. In line with this policy and the current situation in China [16], the core providers of dementia three-level prevention services include dementia-friendly communities (DFCs), community health service centers (CHSCs), and dementia specialist clinics (DSCs). These settings were therefore selected as the primary field sites for this research. The residents and service providers in these settings are key stakeholders in these services and represent the frontline of service delivery. Therefore, this study aimed to explore the programs and challenges in providing dementia three-level services from the perspectives of these key stakeholders, using the RMIC as the guiding framework.

### Research questions

What programs are primarily involved in dementia three-level prevention services?

What are challenges in providing dementia three-level prevention services to residents?

## Methods

### Study Design

Ethnography is about telling credible, rigorous, and authentic stories. It gives voice to people in their own local context, typically relying on verbatim quotations and a thick description of events [17]. This study employed a focused ethnography, which is a recommended method in conducting older people research. In this study, we adopted a multi-site ethnography approach using semi-participatory observations and in-depth interviews, to comprehensively explore the challenges.

### Study Settings

The fieldwork took place at three DFCs, four CHSCs, and two DSCs. The basic characteristics of these sites are described in Supplementary File 1.

### Participants

Purposive and snowball sampling method were combined to select stakeholders for observations and interviews. All interviewees were selected from observed participants. The inclusion and exclusion criteria for participants across different settings were presented in Supplementary File 2. In this study, after observing 24 service providers and 26 residents or family members and conducting interviews with 17 of the participants, no new categories were generated from the data collection and analysis [18]. Therefore, the data collection stopped.

### Theoretical Framework

The RMIC included two scopes (person-focused vs. population-based), four types (system, organizational, professional, and clinical integration) and two enablers (functional and normative integration) [15,19]. In this study, the population-based scope was defined as residents with normal cognition, Mild Cognitive Impairment (MCI) or dementia. The person-focused scope was treated as a foundational norm and was thus incorporated into the dimension of normative integration. Two enablers acted as connectors across processes, facilitating collaboration among professionals, organizations, and systems to deliver integrated care; therefore, they were analyzed alongside the other integration types. We then discussed and identified key elements for each dimension by reviewing literature that had systematically analyzed potential components of the RMIC [10,20,21]. These dimensions and elements were used to design the observation and interview outlines, enabling an exploration of the current state and challenges. Taking “organizational functional position” as an example, empirical questions included: “Which organizations are involved in providing dementia three-level prevention services, such as health education, screening, and diagnosis?”, “What are their specific functions?”, and “How do these organizations coordinate with one another?” The questions were basically designed to be broadly opening, which would prioritize the storytellers’ perspective.

### Data Collection

After obtaining informed consent from site administrators, the researcher collected data in the role of a student nurse at the CHSCs and DSCs, and as a volunteer at the DFCs. Nine administrators of these settings served as key informants to recommend the fields for observations. The study employed the semi-participatory observation method. During this process, the researcher engaged in activities such as dementia health education and screening, while observing and interacting with individuals. The observation began with a descriptive overview of the environment and informal communication to build trusting relationships. After obtaining informed consent from participants, formal observation commenced. The researcher followed service providers during their daily routines to observe their work. Field notes were taken for each observation session, documenting characteristics such as location, time, date, and events. The data were collected through recording and photography, and reflections were written within 24 hours of each session. In total, 59 observation sessions were conducted: 13 sessions in DFCs (nearly 8 hours); 14 sessions in CHSCs (a mix of six 4-hour sessions and eight 8-hour sessions), 32 sessions in DSCs (4 hours each). This amounted to a total of 320 observation hours, generating nearly 90,000 Chinese words of field notes and 50 photographs. In the in-depth interviews, participants were encouraged to guide the conversation’s direction, content, and pace. The researcher listened and gently redirected them if their narratives deviated significantly from the study’s aim. The questions sequence was flexible and adapted to the flow of conversation. All interviews were transcribed verbatim within 24 hours and the transcripts were returned to the interviewee for correction before data analysis. Approximately 140,000 words were transcribed and analyzed.

### Data Analysis

A combination of deductive and inductive analysis was employed to identify the challenges. Both approaches shared a common preparation phase. The researchers immersed themselves in the data by repeatedly reviewing recordings and field notes, transcribing interviews, and segmenting the text into manageable content categories. Keywords, phrases, and sentences related to dementia three-level prevention services were systematically coded. To understand the challenges, all data were reviewed and coded for correspondence with dimensions and elements of the RMIC. Any challenges that did not align with this existing framework were used to generate new categories following inductive analysis principles. The researchers read through the materials, writing notes and headings to generate initial codes. Codes sharing common attributes were then grouped into categories. These categories were subsequently compared and consolidated under higher-order categories through an analysis of their similarities and differences. Data analysis started from choosing the subject to the completion of the manuscript [17]. Data sorting and analysis were performed simultaneously and iteratively. NVivo 12 Plus software was used to facilitate data management.

### Consideration of Rigor

This study employed methodological and investigator triangulation to enhance rigor [22]. First, data were collected through both interviews and observations to ensure comprehensive perspectives. Second, all aspects of the research—including design, data collection, analysis, findings, and interpretations—were carefully reviewed and discussed by the research team to strengthen transparency. Third, preliminary findings and interpretations underwent an external check by various stakeholders. They reviewed the results and provided feedback, ensuring the findings were grounded in reality. To further establish the trustworthiness and validity of the analysis, we incorporated authentic quotations from the data without disclosing participants’ identities.

### Ethics Approval and Consent to Participate

This study was approved by the Research Ethics Committee of Soochow University, and was conducted in accordance with the ethical standards outlined in the Declaration of Helsinki.

Service providers were initially identified for observation based on predefined criteria and recommendations from key informants. We began formal observation and interviews only after informing these providers about the research purpose, data collection methods, their right to withdraw, and obtaining their informed consent. During observations, residents who met the inclusion criteria were invited to participate. For residents with dementia in DSCs, we obtained full informed consent from their family members. For residents approached in DFCs, informed consent was obtained directly from them after they received a complete explanation of the study. This manuscript contains no personally identifiable information. A draft was reviewed by administrators of the participating sites, who granted permission for its publication. All participants understood that their participation was voluntary and that they could withdraw consent at any time without their rights being affected.

## Results

### Participant Characteristics

A total of 50 participants were observed, including 24 service providers and 26 residents or family members. Seventeen of the participants were also interviewed. Their characteristics are presented in Supplementary File 3–7.

### Dementia Prevention Programs

**Figure 1:**
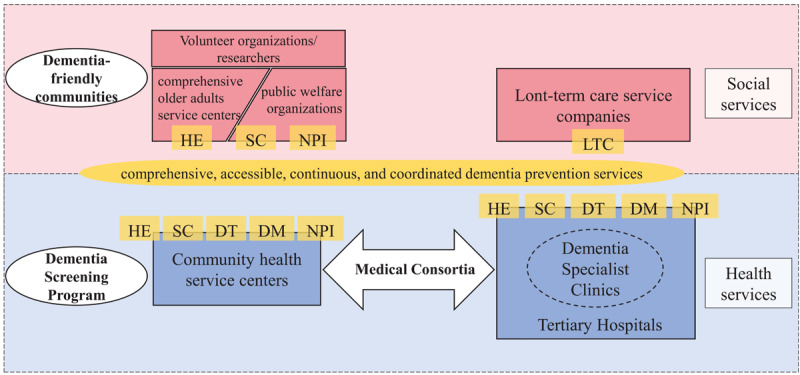
Service bodies, their relationships, and their roles in dementia three-level prevention services. Note: (1) Abbreviations: HE, health education; SC, screening; DT, diagnosis and treatment; DM, Disease Management; NPIs, non-pharmacological interventions; LTC, long-term care. (2) Dementia-friendly communities are inclusive environments designed to empower individuals with dementia and their families to live well, participate in meaningful activities, and contribute to community life. Core services include HE, SC, and NPIs. (3) Community health service centers are grassroots health institutions that provide basic public health services and medical services in the community, mostly public non-profit institutions. They operate under government administration with guidance from higher-level health authorities. (4) Tertiary hospitals are large-scale medical institutions that provide specialized healthcare services and bear responsibilities for medical education and scientific research across multiple regions. (5) Medical Consortia are collaborative networks of healthcare institutions that integrate resources to provide HE, SC, DT, DM and NPIs. (6) Long-term care service companies are organizations specializing in the provision of long-term care services.

The programs involved in dementia three-level prevention services are presented in Supplementary File 8. They included Dementia Screening Program, DFCs, Medical Consortia and Long-term Care Services. Figure 1 illustrates the service bodies, their relationships, and their roles in dementia three-level prevention services.

### The Challenges

This study identified 32 challenges in the provision of dementia three-level prevention services from the perspective of the RMIC. These challenges encompassed four at the system level, ten at the organizational level, seven each at the professional and clinical levels, and four additional challenges, presented in Supplementary File 9.

### System Integration

**Challenge 1: Lack of formal task plans from administrative organization**. The work of neighborhood committees was typically directed by higher-level administrative bodies. The failure to disseminate relevant notifications to some committees resulted in skepticism regarding their participation in primary prevention work.

I followed V4 to the neighborhood committee, but the staff there appeared unaware of the program. (Observation, DFC3)

**Challenge 2: Dementia not involved in health management**. Dementia has not been incorporated into the health management scope. Although China has recommended that regions with conditions should include cognitive screening in basic public health services, most CHSCs implemented such screening only in a cursory manner.

“That’s an item in physical examinations. Since residents can come here by themselves, go to the right department and answer questions independently, we assume their cognitive ability is normal.” It is not reasonable to conclude that residents have normal cognitive function based solely on these criteria. (Interview and reflections, S12)

**Challenge 3: Ineffective implementation of family physician system**. Due to residents’ unwillingness, CHSCs staff could only secure contracts with a limited number of willing individuals, which failed to adequately serve those most in need, particularly residents with MCI or dementia.

S5 brought a woman approximately 40 years old to sign a family doctor contract. (Observation, CHSC3)

**Challenge 4: Uncharged non-pharmacological interventions (NPIs)**. The healthcare system does not allow for the billing of NPIs, and no sustainable funding mechanisms were available to support implementation of NPIs.

“Although specialists expressed interest in providing cognitive training, they did not actually offer it. We proposed packaging these interventions into prescribable formats, which would allow our departments to generate revenue. Providing these services for free was seen as unsustainable.” (Interview, Y6)

### Organizational Integration

**Challenge 1: Unformed atmosphere of collaboration**. Primary prevention efforts, particularly screening, were difficult to implement through CHSCs alone. Both leadership and healthcare providers demonstrated limited awareness of the need to mobilize broader social resources.

“Screening cannot rely solely on us, and it can be carried out by trained personnel, such as volunteers.” Previous interviews and observations revealed that three CHSCs faced the same problem, but S11 was the first to propose this idea. (Interview and reflections, S11)

**Challenge 2: Inadequate engagement of neighborhood committees**. These committees hold a unique authority in mobilizing residents for primary prevention services. However, their reluctance or limited involvement in coordinating residents’ participation significantly hindered the implementation of services.

Before the screening, V2 told us that the neighborhood committee was not being proactive and we had to find residents by ourselves. Consequently, only a small number of residents participated in the afternoon session. (Observation, DFC2)

**Challenge 3: Verbal agreements**. Clarifying respective goals, interests, and tasks in signed contracts could facilitate efficient and smooth project progress. However, cooperation based solely on verbal agreements created ambiguity for the research team regarding implementation, thereby impeding effective collaboration.

“Without a set of rules and regulations established early on, implementation efficiency will suffer in later stages, and screening quality will be difficult to guarantee.” (Interview, Y5)

**Challenge 4: Inappropriate values**. Leaders’ inappropriate values (only pursuing plan completion, submitting reports, and being unwilling to ‘trouble’ other stakeholders, etc.) directly hindered the linkage with related resources.

V3, two volunteer researchers, and staff from a technology company attended a screening preparation meeting. When I asked about coordinating with the neighborhood committees, V3 stated, “It’s not good to trouble others.” (Observation, DFC3)

**Challenge 5: Residents’ distrust**. Residents and their families lacked trust in the screening results and referral recommendations, and therefore did not seek further services.

“If you suggest that these individuals seek medical treatment, they may feel that we have other purposes.” (Interview, S8)

**Challenge 6: Lack of referral**. Social and primary healthcare service institutions seldom referred residents with suspected cognitive impairment to tertiary hospitals for diagnostic and treatment.

There was no effective connection between the initial screening and the subsequent care. Residents identified with potential issues during screening did not receive corresponding diagnosis and treatment. (Observation, DFC1 and DFC2)

**Challenge 7: Absence of common goals and benefits-sharing mechanisms**. Common goals and corresponding benefits-sharing mechanisms between different levels of care were not formed. CHSCs often struggled to make accurate diagnoses. Some general practitioners chose to do nothing, while those from well-developed CHSCs sometimes prioritized empirical diagnosis and treatment rather than referring residents to specialized clinics. Residents in stable conditions frequently bypassed CHSCs to seek further services at tertiary hospitals.

“There seems to be some initial ideas during the planning phase, such as transferring unstable residents upstream and stable ones downstream. However, after I refer patients to tertiary hospitals, what about the follow-up? Why should specialists transfer patients back? They also need to maintain their patient volume. Moreover, some medications are still not available here.” (Interview, S8)

Once residents received their initial consultation, they usually chose to continue their follow-up here. (Observation, DSC1)

**Challenge 8: Inadequate resource sharing**. There was inadequate resource sharing among institutions within the Medical Consortia, such as the first-line medication and health information of residents with dementia.

We only have common drugs here, but more advanced drugs need to be obtained from tertiary hospitals. (Observation, CHSC3)

“The information system sometimes just doesn’t work well. We’re supposed to be able to access medical records from referral hospitals. Sometimes we can, but not every time.” (Interview, S9)

**Challenge 9: Uncoordinated functional positions**. Most CHSCs prioritized the management of chronic conditions such as hypertension and diabetes, while cognitive impairments received limited attention. A few well-developed CHSCs engaged in provisional diagnosis and treatment but did not provide follow-up care. In contrast, DSCs were primarily responsible for diagnosis, treatment, and follow-up.

“We basically do not engage in the diagnosis, treatment, and management of cognitive-related conditions here.” (Observation, CHSC1 and CHSC2)

There was an average of 1–2 first-visit residents with cognitive-related conditions in DSC1, and 2–4 residents in DSC2 each day, with the majority of attendees returning for follow-up. (Observation, DSC1 and DSC2)

**Challenge 10: Unformed cross-institutional dementia management team**. Residents relied more on their family members and DSCs for care and management. Staff at CHSCs and specialists in tertiary hospitals did not establish cross-institutional collaborations to implement treatment and management.

The cross-institutional team was formed among neurologists, general practitioners, and ward nurses. However, it mainly served post-stroke residents, and there was no team for dementia. (Observation, CHSC4)

### Professional Integration

**Challenge 1: Limited human resources**. CHSCs needed to mobilize and allocate their limited clinical staff, including physicians, nurses, and other health technicians, to conduct the screenings while simultaneously managing other routine responsibilities, which frequently resulted in staffing shortages.

“The screening was clearly understaffed. Some residents responded relatively slowly, and the task was huge, so we often achieved very little in the end.” (Interview, S1)

“Honestly, it wasn’t realistic to assign someone exclusively to this task because our outpatient was quite busy.” (Interview, S6)

**Challenge 2: Underutilization of professional expertise**. While service providers collaborated with dementia specialists and research teams, the professionals’ guidance in scientifically and systematically designing primary services remained underutilized.

This underutilization created constraints in several areas, such as modifying screening questionnaires, obtaining complete datasets for deeper analysis, and establishing connections with other community resources. (Observation, DFC3)

**Challenge 3: Shortage of volunteers**. While DFCs staff participated in primary prevention work, their capacity is limited. Trained volunteers were crucial for providing support, but recruiting an adequate number remained a challenge.

When asked if there were enough volunteers, V2 stated, “No. Having two volunteers during your observation is actually a very good situation. Most of the time, we can only rely on ourselves.” (Observation, DFC2)

**Challenge 4: Lack of motivation**. Service providers, including nurses and general practitioners, lacked motivation to deliver primary prevention services.

This work was not implemented, as dementia was not included within the health management scope. Furthermore, nurses were occupied with therapeutic work, such as drawing blood. (Observation, CHSC1~4)

**Challenge 5: Underutilized potential for healthcare professionals**. Healthcare professionals did not fulfill their potential in services. Physicians focused more on health education for common chronic diseases and often lacked the capacity to perform dementia diagnosis, treatment, and management. Nurses were assigned to tasks such as treatment, care, and blood collection, in wards.

General practitioners in CHSC3 expressed that they could implement dementia-related health education, screening, etc., which implied that such competencies could be developed in practice. (Observation, CHSC3)

By contrast, CHSC1 and 2 basically did not implement dementia health education, screening, diagnosis, treatment, or management. (Observation, CHSC1 and 2)

**Challenge 6: Insufficient capability**. General practitioners, nurses, and other related healthcare professionals lacked adequate knowledge and skills to conduct dementia-related health education, screening, and NPIs.

S4: “We general practitioners can give lectures about hypertension and diabetes, but when it comes to dementia, our knowledge is relatively limited.” (Interview, S4)

When asked about their familiarity with NPIs for dementia, some healthcare workers reported that they didn’t know. (Observation reflections, CHSC1~4)

**Challenge 7: Incomplete team structure**. The multidisciplinary teams in tertiary hospitals generally included neurologists, psychologists, radiologists, and other professionals, enabling the provision of systematic dementia diagnosis and treatment. However, the lack of nurses hindered the implementation of NPIs and ongoing management. Besides, the assignment of non-specialist physicians, reduced their role to a formality. Incomplete team structure made Medical Consortia failed to fulfill their intended function.

“Many specialized nursing roles have been well developed, such as diabetes specialized nurses. For nurses in the neurology department, their expertise lies more in stroke treatment and care than in dementia.” (Interview, Y5)

“Many residents are actually unfamiliar with Medical Consortia, so they still go to tertiary hospitals if they have health issues.” (Interview, S11)

### Clinical Integration

**Challenge 1: Difficulty in screening**. Service providers perceived that it was difficult to complete screening during physical examinations due to the time-consuming screening tools, age-related declines in residents’ hearing, and constraints in human resources.

“Some residents are older and their hearing and vision are not very good. Overall, it is quite difficult to conduct the screening.” (Interview, S9)

“It is necessary to develop simplified scales with good sensitivity and specificity.” (Interview, S5)

**Challenge 2: Unavailable NPIs**. NPIs for residents with dementia, such as exercising, cognitive training, and other activities, were typically dependent on training programs and the efforts of family caregivers. These interventions were not systematically implemented within clinical settings, including CHSCs and tertiary hospitals.

“I usually encourage her to socialize more and exercise appropriately.” (Interview, J1)

**Challenge 3: Lack of comprehensive assessment, goals, and care plans**. The comprehensive assessment was not conducted, which led to a lack of goal setting and care plans. Therefore, insufficient attention was given to social support and available care service resources.

Residents with cognitive impairment typically received assessments including activities of daily living, cognitive function, and depression scales during diagnosis and treatment. (Observation, DSC1 and DSC2)

**Challenge 4: Delays in accessing long-term care services**. While long-term care companies typically promoted their services through community outreach and DSCs, some eligible residents still failed to receive timely care.

L7 experienced difficulty walking as early as February 2022 and learned about long-term care services in June 2022. When visiting the doctor in July, J7 requested that the disability be documented in the medical record. However, the family was informed that they would need to wait six months to become eligible for care services. (Observation, DSC1)

**Challenge 5: Unmet personalized long-term care needs**. The implementation of long-term care services failed to address residents’ personalized needs, such as bathing preference.

“The nursing aides here told me that residents are required to wear shorts while showering. If so, what is the point of taking a shower?” (Interview, J7)

**Challenge 6: Standardized process of long-term care**. Regions and service companies have established standardized long-term care protocols to protect patient privacy and ensure caregiver occupational safety, which cannot adequately address individual needs.

Consultation with leaders of long-term care companies confirmed that standardized service processes were adopted to safeguard resident privacy and staff safety. However, these protocols could incorporate greater flexibility to accommodate the preferences of residents and families—for instance, by allowing cleaning of private areas under family members’ supervision. (Interview reflections, J7)

**Challenge 7: Low level of active participation of residents and their families**. Engagement in services remained insufficient, primarily manifesting as reluctance or passive involvement in health education and screening activities, delayed medical consultation after suspicious symptoms, and poor adherence to treatment and management following diagnosis.

“If you want them to fill out questionnaires, you need to provide small gifts. Otherwise, they won’t participate.” (S1, Interview)

### Additional Challenges

**Challenge 1: Lack of dementia-related knowledge**. Residents and their families demonstrated limited understanding of dementia prevention, including its risk factors, early symptoms, and management strategies for associated psychological and behavioral symptoms.

C5 stated, “Maintaining a good mood and talking to people are the most important.” (Observation, DFC3)

Y1 said, “They doesn’t feel painful in the early stages. I advised them to return for a clinical examination after going home, but many of them would not come.” This comment was based on her experience with such cases. (Observation, DSC2)

“When she lay in bed, she sometimes talked to herself as if speaking to someone real. I didn’t know how to respond.” (Observation, J2)

**Challenge 2: Stigma**. The dementia-related stigma led residents or their family members to avoid participating in health education and screening activities, deny the condition, or repeatedly seek diagnoses from different hospitals.

L1 and her spouse sought medical treatment from hospitals in Suzhou, Shanghai, and other regions. They were unwilling to acknowledge that L1 had dementia until her cognitive impairment had progressed to a severe stage. (Observation, DSC2)

**Challenge 3: Difficulty in caring work**. Residents with moderate to severe cognitive impairment basically lost the ability to care for themselves. When caregivers were older and had limited physical capacity themselves, they struggled to manage the arduous and demanding tasks of daily care.

“He was eating, and the rice and vegetables fall on his clothes. He didn’t even know how to get dressed. When I handed him pants, he put both feet into the same leg of his trousers. It’s exhausting.” (Interview, J7)

**Challenge 4: Lack of professional and convenient day care institutions**. Individuals with MCI or dementia required care from trained professionals, yet there was a notable absence of specialized, community-based institutions to provide such support.

“My life has completely changed, and everything has changed. I no longer have my own life. I have to follow her and can’t go out. It would be ideal to have an institution nearby to provide her with care.” (Interview, J8)

## Discussion

This study investigated key programs involved in dementia three-level prevention services and their challenges. Four programs were identified: Dementia Screening Programs, DFCs, Medical Consortia, and Long-term Care Services. Furthermore, 32 challenges were categorized across integration levels: four at the system level, ten at the organizational level, seven each at the professional and clinical levels, and four additional challenges.

### Enriching Diversity of Programs and Strengthening Primary Prevention Services

Of the four programs examined in our study, the Dementia Screening Program was implemented as a temporary, locally-specific initiative, whereas the other three were more routinely established. In contrast, the UK’s Prime Minister’s Challenge on Dementia 2020 outlined over 50 specific commitments across four core themes—risk reduction, health and care, awareness and social action, and research [23]. This national initiative encompasses a range of programs, such as the NHS Health Check, One You, the Brain Age tool, Dementia Friends, DFCs, the Care Act, and the “Well Pathway for Dementia” series. Compared to the UK, service provision in our region reveals notable gaps, primarily in two aspects. First, there is limited diversity in service content. Second, current efforts remain predominantly focused on screening, diagnosis, and treatment, with relatively underdeveloped services for primary prevention. To address these shortcomings, policymakers and research institutions should prioritize enriching the diversity of programs and strengthening primary prevention services.

Our field research indicates that the implementation of these four main services continues to encounter multiple level challenges, spanning from micro-level to macro-level. It is therefore imperative for policymakers and researchers to prioritize the development of targeted strategies addressing these challenges.

### Integrating Resources from Multiple Parties for Temporary Initiative

The Dementia Screening Program was a temporary, localized initiative centered on screening, and it encountered challenges from multiple levels. The most fundamental of these was the unformed atmosphere of collaboration, which resulted in limited human resources and further difficulty in screening. This finding aligns with Gong’s study [24], which identified insufficient time as a key barrier in screening. In our context, limited human resources and insufficient time were interrelated issues that collectively impeded screening and management processes. Besides, our field research further revealed that CHSCs leaders currently lack adequate awareness of collaborative practice, which may contribute to downstream issues. Thus, it is crucial for them to prioritize integrating resources from multiple stakeholders to support service implementation.

### Improving DFCs Services Across Multiple Levels

The DFCs primarily faced challenges at the system, organizational and professional levels.

#### Developing Administrative Plans and Coordinating Relevant Organizations

At the system level, the DFCs lacked formal task plans from administrative organization. In China, DFCs represent a common form of social service that provides dementia-related health education, screening, and resource coordination. For instance, Shanghai launched its fifth batch of DFCs construction projects in 2023 [25], while Suzhou initiated a DFC pilot program in October 2022. In practice, the system challenge, i.e., the absence of clear administrative plans, led to organizational challenge, i.e., inadequate engagement of neighborhood committees. By contrast, the United Kingdom, which has implemented successful DFC programs, emphasizes governmental involvement and leadership to improve service delivery, as outlined in the Prime Minister’s Challenge on Dementia 2020 [23]. Therefore, the Civil Affairs department, as the regulatory authority, should take an active role in formulating formal administrative task plans and disseminating them to relevant organizations and personnel. This would help ensure the projects’ legitimacy and effectively mobilize stakeholders to participate.

#### Fostering a Collaborative Mindset and Formalizing Written Agreements

Challenges at the organizational level, such as inappropriate values, unformed atmosphere of collaboration, contributed to difficulties at the professional level, such as underutilization of professional expertise and a shortage of volunteers, and at the clinical level, such as challenges in screening. Therefore, strategies targeting the organizational level form the foundation for addressing issues at other levels. First, inappropriate values represented a key organizational challenge. The Alzheimer’s Disease International regards partnerships as an important element of DFCs [26]. However, some DFC leaders hold attitudes such as “It is not good to trouble others,” which conflict with the collaborative and socially driven nature of DFCs. A collective commitment to this cause and working in collaboration and partnership is critical [26]. Thus, DFC leaders should adopt a collaborative mindset and leverage the advantages of accessible institutions and professionals. Second, verbal agreements can lead to the underutilization of professional expertise. Verbal agreements often fail to establish clear consensus on critical aspects such as responsibilities and benefits-sharing, thereby hindering effective collaboration. Based on our findings, DFCs leaders should establish written agreements with partners to improve the service provision.

#### Addressing Knowledge Gaps and Stigma to Enhance Participation

A major challenge for DFCs was the low level of active participation from residents and their families at the clinical level, compounded by limited knowledge of dementia and stigma. Residents and their families demonstrated limited knowledge of dementia, a finding consistent with previous research [5]. We further observed that even caregivers with professional backgrounds, such as J2, lacked adequate knowledge and the necessary skills to deliver appropriate care. Overall, this knowledge gap often discouraged residents and their families from participating in health programs and seeking further services, in part due to the stigma. Therefore, systematic dementia education and stigma-reduction efforts are urgently needed to enhance service participation among residents and their families.

### Strengthening Medical Consortia Across Multiple Levels

Medical Consortia primarily faced challenges at the system, organizational, professional and clinical levels.

#### Strengthening Health Management, Implementing Family Physician System, and Improving Financing and Payment of NPIs

Medical Consortia faced four challenges at the system level. The first major challenge was lack of dementia in health management, which could lead to organizational challenges, and further led to downstream challenges. In China, CHSCs undertake health management programs for older adults, including physical examinations and disease health management. Ideally, these annual check-ups could detect cognitive impairments early and identify a sufficient number of potential service recipients for Medical Consortia. In practice, however, the screening tools commonly used often only identify dementia at a relatively advanced stage. This gap may be attributed to the fact that dementia screening is merely recommended rather than mandated in national health management policy. By contrast, in the United Kingdom, the NHS Health Check includes a mandatory dementia awareness component for individuals aged 65 and above [23], which helps strengthen dementia prevention efforts. Therefore, relevant departments should advocate for standardized dementia screening tools and management guidelines, and establish more supportive conditions to advance dementia health management [24]. Second, system-level challenges included ineffective implementation of family physician system and uncharged NPIs, which subsequently created difficulties at the organizational and clinical levels. The availability of family physicians is crucial for dementia management. However, our observations indicated that the family physician system had not yet been fully implemented, which aligns with a previous study [27]. Many factors may influence its implementation, including patient-provider matching mechanisms, community care quality, and familiarity with family physicians, etc [27,28]. Besides, the fact that NPIs were uncharged made them unavailable in practice, which aligns with a study in Australia [29]. Therefore, governments should strengthen the implementation of the family physician system and improve the financing and payment of NPIs to mitigate downstream challenges.

#### Establishing Common Goals and Benefits-Sharing Mechanisms, and Strengthening Residents’ Trust

Medical Consortia faced six challenges at the organizational level. First, the absence of common goals and benefits-sharing mechanisms represented the root challenge, which could lead to other organizational and professional level challenges, thus should be treated as priorities. The absence of common goals and benefits-sharing mechanisms among institutions prevent them and their professionals from fulfilling their roles, participating actively, and delivering related services—a pattern also observed in nursing home settings [30]. Thus, policymakers and researchers should focus on developing strategies for institutional common goals and benefits-sharing mechanisms. Second, our study found that residents’ distrust undermined referral processes both within Medical Consortia and from DFCs to DSCs, resulting in the further services discontinued. A previous study indicated that distrust impacted the acceptance of integrated social care services [31], which aligns with our finding. We also observed that distrust hindered effective care coordination across different levels of healthcare institutions. This issue arises from multiple factors, including the involvement of third-party companies in implementing DFCs and the limited capacity of CHSCs [24,28]. Therefore, targeted efforts are needed to foster and strengthen trust in primary care services.

#### Increasing Providers’ Capability and Improving Team Structure

Medical Consortia faced four challenges at the professional level. First, the limited capability of professionals within CHSCs impeded effective dementia screening and management. A study corroborates that general practitioners at CHSCs often lack proficiency in essential areas such as health education, screening, and dementia care [32]. Supporting this, research from Singapore found that while physicians recognized the importance of early detection and held positive attitudes toward diagnosis, they reported low confidence in diagnosing dementia, as well as in communicating with and managing care for people with dementia [33]. Consequently, strengthening training programs to elevate providers’ capability is imperative. Second, the absence of dementia specialist nurses hindered the implementation of NPIs and the management of residents with dementia. Nurses’ knowledge and care approaches are fundamental to ensuring quality and improving patients’ lives; however, evidence suggests that even outpatient nurses in tertiary hospitals possess limited dementia-related knowledge [34]. Another missing key role was non-specialist physicians assigned in Medical Consortia, which created a mismatch between treatment needs and available expertise. Therefore, institutions involved in Medical Consortia must prioritize improving team structure to enhance the accessibility and quality of dementia-specific services.

### Reducing Waiting Periods, Meeting Personalized Needs, Connecting Caregivers to Support, and Establishing Day Care Institutions for Long-term Care Services

The long-term services faced clinical and additional challenges. First, delays in accessing care and an over-reliance on standardized care processes presented key challenges, with the latter frequently resulting in residents’ unmet personalized needs. These challenges were observed during residents’ visits to DSC settings. High-quality long-term care depends on recognizing residents as individuals and adapting care to their specific needs and preferences [35]; thus, promoting long-term care services should not come at the expense of addressing residents’ personal requirements. Therefore, policymakers could consider reducing the mandatory waiting period for long-term care eligibility, and providers should strive to balance standardized services with respect for the personalized needs of residents and their families. Second, older caregivers faced difficulty in the caring work. Previous research has demonstrated that caregivers often report poorer self-rated health and higher levels of anxiety and depression [36]. It is therefore essential for service providers to proactively connect caregivers with available support services. Finally, family members also reported a lack of professional and convenient care institutions. During the field research, DFC3 attempted to provide daytime care for residents with dementia, but failed to sustain them due to inadequate care capacity and high operational costs. This challenge is common in many countries, including England [37], which is considered one of the best places for people with dementia. To address this issue, relevant departments should provide policy and economic support to strengthen the establishment of dementia day care institutions.

### Limitations

This study has a limitation regarding site selection. Initially, we aimed to include rural observation sites. However, due to the scarcity of dementia prevention programs in these areas, we ultimately selected institutions in the first-tier cities of Shanghai and Suzhou, where such projects were actively being implemented. Consequently, the identified challenges and corresponding strategies are primarily applicable to well-developed urban contexts.

## Conclusions

This one-year ethnographic study identified four programs in dementia three-level prevention services and related challenges of implementing these services in China from an integrated care perspective. Enriching diversity of programs and strengthening primary prevention services will be beneficial for improving the dementia three-level services. Besides, identified 32 challenges can offer valuable insights for researchers, policymakers, and other stakeholders to develop targeted interventions, inform policy, and optimize service delivery in dementia three-level prevention services, ultimately enhancing their comprehensiveness, accessibility, continuity, and coordination.

## Additional Files

The additional files for this article can be found as follows:

10.5334/ijic.9106.s1 Supplementary File 1. Characteristics of observation sites.

10.5334/ijic.9106.s2 Supplementary File 2. The inclusion and exclusion criteria of observed and interviewed service providers and recipients from different settings.

10.5334/ijic.9106.s3 Supplementary File 3. Characteristics of residents from Dementia-friendly communities (n = 10).

10.5334/ijic.9106.s4 Supplementary File 4. Characteristics of service providers from Dementia-friendly communities (n = 5).

10.5334/ijic.9106.s5 Supplementary File 5. Characteristics of service providers from Community health service centers (n = 14).

10.5334/ijic.9106.s6 Supplementary File 6. Characteristics of service providers from Dementia specialist clinics (n = 6).

10.5334/ijic.9106.s7 Supplementary File 7. Characteristics of older adults and their family members from Dementia specialist clinics (n = 16).

10.5334/ijic.9106.s8 Supplementary File 8. The programs involved in dementia three-level prevention services.

10.5334/ijic.9106.s9 Supplementary File 9. The identified challenges through rainbow model of integrated care.

## Data Availability

The Chinese datasets analyzed in the current study are available on reasonable request from the first author.
