# Supplementary material for: The Challenges in Dementia Three-Level Prevention Services Through Integrated Care Theory: Insights from an Ethnographic Study
Source: Int J Integr Care. 2026 Aug 10;26(3):8. doi: 10.5334/ijic.9106 (PMC13475528; doi:10.5334/ijic.9106)
Supplement: Supplementary file 1 [file ijic-26-3-9106-s1.pdf]

Supplementary File 1. Characteristics of observation sites

Code	Location
DFC1	● It was located in Shanghai.<br>● It was primarily implemented by a public welfare organization focused on brain health, with a commitment to dementia prevention and early intervention. It undertook dementia friendly community projects several times.<br>● It was primarily applied for by the street administrative organization and implemented by the public welfare organization.<br>● It carried out health education, early screening, intervention, and care support for dementia.
DFC2	● It was located in Shanghai.<br>● It was primarily implemented by the community's comprehensive older adults service center, which was committed to building a one-stop service platform. This platform integrated dining, accommodation, recreation, health care, and elderly care, providing diversified and multi-level services for residents in the surrounding communities.<br>● It was primarily applied for by the street administrative organization and implemented by the comprehensive older adults service center.<br>● It carried out health education, early screening, intervention, and care support for dementia.
DFC3	● It was located in Suzhou.<br>● It was primarily implemented by the community's comprehensive older adults service center, which had eight functional service areas, including professional care, smart elderly care, nutritional services, and medical health care.<br>● It was primarily applied for and implemented by the center.<br>● It carried out health education, early screening, intervention, and care support for dementia.
CHSC1	● It was located in Suzhou.<br>● Its total area was approximately 3,000 square meters.<br>● It was equipped with departments such as general medical care, pharmacy, nursing, traditional Chinese medicine, as well as a dedicated outpatient service affiliated with a Medical Consortium.<br>● It served seven neighborhood committees, providing services to nearly 40,000 registered residents in the communities.<br>● It provided the national basic public health services, which included health education, vaccination, child health care, establishment of residents' health records, chronic disease patient management, and traditional Chinese medicine health management.<br>● It operated under an integrated "Six-in-One" model that combined medical treatment, prevention, health care, rehabilitation, health education, and family planning technical guidance.<br>● It established Medical Consortia partnership with tertiary hospitals.<br>● It previously implemented Suzhou's "531" project, which included the screening and

Code	Location
	prevention of cardiovascular and cerebrovascular diseases, osteoporosis, and other chronic conditions.
CHSC2	● It was located in Suzhou.<br>● Its total area was approximately 1,800 square meters.<br>● It served 15 neighborhood committees, providing services to nearly 60,000 registered residents in the communities.<br>● It provided the national basic public health services, which included health education, vaccination, child health care, establishment of residents' health records, chronic disease patient management, and traditional Chinese medicine health management.<br>● It operated under an integrated "Six-in-One" model that combined medical treatment, prevention, health care, rehabilitation, health education, and family planning technical guidance.<br>● It established Medical Consortia partnership with tertiary hospitals.<br>● It previously implemented Suzhou's "531" project, which included the screening and prevention of cardiovascular and cerebrovascular diseases, osteoporosis, and other chronic conditions.
CHSC3	● It was located in Suzhou.<br>● Its total area was approximately 3,500 square meters.<br>● It served 12 neighborhood committees, providing services to nearly 60,000 registered residents in the communities.<br>● It provided the national basic public health services, which included health education, vaccination, child health care, establishment of residents' health records, chronic disease patient management, and traditional Chinese medicine health management.<br>● It operated under an integrated "Six-in-One" model that combined medical treatment, prevention, health care, rehabilitation, health education, and family planning technical guidance.<br>● It established Medical Consortia partnership with tertiary hospitals.<br>● It previously implemented Suzhou's "531" project, which included the screening of cognitive impairment.
CHSC4	● It was located in Shanghai.<br>● Its total area was approximately 10,000 square meters.<br>● It served one neighborhood committee, providing services to nearly 90,000 registered residents in the communities.<br>● It provided the national basic public health services, which included health education, vaccination, child health care, establishment of residents' health records, chronic disease patient management, and traditional Chinese medicine health management.<br>● It operated under an integrated "Six-in-One" model that combined medical treatment, prevention, health care, rehabilitation, health education, and family planning technical guidance.<br>● It established Medical Consortia partnership with tertiary hospitals.

Code	Location
DSC1	● It was a geriatric medicine clinic from a tertiary hospital, staffed by neurology specialists.<br>● It specialized in the diagnosis, treatment, and management of dementia, including Alzheimer's disease and vascular dementia.<br>● Outpatients came for either memory/cognitive complaints or other cerebrovascular issues.
DSC2	● It was a memory clinic from a tertiary hospital, staffed by neurology specialists.<br>● It specialized in the diagnosis, treatment, and management of dementia, including Alzheimer's disease and vascular dementia.<br>● Most outpatients came for memory and cognitive issues.

Note: DFCs, Dementia-friendly communities; CHSCs, Community health service centers; DSCs, Dementia special clinics.
