# Supplementary material for: The Challenges in Dementia Three-Level Prevention Services Through Integrated Care Theory: Insights from an Ethnographic Study
Source: Int J Integr Care. 2026 Aug 10;26(3):8. doi: 10.5334/ijic.9106 (PMC13475528; doi:10.5334/ijic.9106)
Supplement: Supplementary file 2 [file ijic-26-3-9106-s2.pdf]

Supplementary File 2. The inclusion and exclusion criteria of observed and interviewed service providers and recipients from different settings

Settings	Participants	Inclusion criteria	Exclusion criteria
DFCs<br>CHSCs<br>DSCs	Observed service providers	(1) Engaged in dementia three-level prevention services, including health education, screening, diagnosis, treatment, etc.;<br>(2) Voluntarily participated with informed consent.	—
DFCs<br>CHSCs<br>DSCs	Interviewed service providers	(1) Engaged in dementia three-level prevention services, including health education, screening, diagnosis, treatment, etc.;<br>(2) Voluntarily participated with informed consent;<br>(3) Minimum six months of work experience with dementia;<br>(4) Effective communication skills.	(1) Inability to provide effective answers as per interview outlines.<br>(2) Withdrawal from the study midway.
DFCs	Observed residents	(1) Residents that participated in the health promotion, screening activities;<br>(2) Voluntarily participated with informed consent.	—
CHSCs	Observed residents	(1) Residents that came for consultation with memory related problems;<br>(2) Voluntarily participated with informed consent.	—
DSCs	Observed residents and their family members	(1) Residents that came for diagnoses or treatment with MCI, Alzheimer's disease, or vascular dementia, and family members that accompanied residents;<br>(2) The observed residents and family members voluntarily participated with informed consent.	—
DSCs	Interviewed family members	(1) Family members of residents diagnosed with MCI, Alzheimer's disease, or vascular dementia;<br>(2) Actively involved in care activities for the residents;<br>(3) Good communication abilities;<br>(4) Voluntarily participated with informed consent.	(1) Inability to provide effective answers as per interview outlines.<br>(2) Withdrawal from the study midway.

Note: DFCs, Dementia friendly communities; CHSCs, Community health service centers; DSCs, Dementia special clinics; "—" indicated that there were no exclusion criteria.
