# Supplementary material for: The Challenges in Dementia Three-Level Prevention Services Through Integrated Care Theory: Insights from an Ethnographic Study
Source: Int J Integr Care. 2026 Aug 10;26(3):8. doi: 10.5334/ijic.9106 (PMC13475528; doi:10.5334/ijic.9106)
Supplement: Supplementary file 3 [file ijic-26-3-9106-s3.pdf]

Supplementary File 3. Characteristics of residents from Dementia-friendly communities (n=10)

Code	Age	Gender	Education	Marriage	Dementia prevention measures that they knew	Chronic disease
A1	62	Male	Technical secondary school	Married	Good mood	No
A2	62	Female	Junior high school	Married	None	Hypertension
A3	70	Female	Illiteracy	Married	None	Hypertension, Coronary heart disease, Osteoporosis
A4	79	Female	Primary school	Married	Contact and communicate with others	Hypertension
A5	63	Female	Junior high school	Married	Good mood，Contact and communicate with others	Hypertension, Diabetes, Dyslipidemia
A6	85	Male	Technical secondary school	Widow	Contact and communicate with others，Family Companion, No smoking	Hypertension, Dyslipidemia, Coronary heart disease
A7	72	Female	Primary school	Married	Contact and communicate with others	Hypertension, Diabetes
A8	73	Female	Primary school	Married	Contact and communicate with others	Hypertension
A9	72	Female	Primary school	Married	Contact and communicate with others	Hypertension, Diabetes, Dyslipidemia
A10	74	Female	Technical secondary school	Married	Contact and communicate with others，Travelling	Hypertension, Joint diseases, Gallstones
