# Supplementary material for: The Challenges in Dementia Three-Level Prevention Services Through Integrated Care Theory: Insights from an Ethnographic Study
Source: Int J Integr Care. 2026 Aug 10;26(3):8. doi: 10.5334/ijic.9106 (PMC13475528; doi:10.5334/ijic.9106)
Supplement: Supplementary file 4 [file ijic-26-3-9106-s4.pdf]

Supplementary File 4. Characteristics of service providers from Dementia-friendly communities (n=5)

Code	Gender	Age	Education	Institution	Position	Dementia work experience	Interview time
V1*	Male	48	Bachelor	DFC1	Person-in-charge	10 years	30 minutes
V2	Male	27	Master	DFC2	Person-in-charge	1.5 years	NA
V3	Male	36	Bachelor	DFC3	Person-in-charge	1.5 years	NA
V4	Female	33	Bachelor	DFC3	Social worker	1 year	NA
Y5*	Female	25	PhD Candidate	DFC3, and DSC2	Volunteer, researcher	2 years	60 minutes

Note: * indicated that they were also interviewed; NA, not available.
