# Supplementary material for: The Challenges in Dementia Three-Level Prevention Services Through Integrated Care Theory: Insights from an Ethnographic Study
Source: Int J Integr Care. 2026 Aug 10;26(3):8. doi: 10.5334/ijic.9106 (PMC13475528; doi:10.5334/ijic.9106)
Supplement: Supplementary file 5 [file ijic-26-3-9106-s5.pdf]

Supplementary File 5. Characteristics of service providers from Community health service centers (n=14)

Code	Gender	Age	Education	Institution	Position	Dementia work experience	Interview time
S1	Female	47	Bachelor	CHSC1	Head nurse	4 years	20 minutes
S2*	Female	38	Bachelor	CHSC1	General practitioner	5 years	10 minutes
S3*	Female	34	Bachelor	CHSC2	Head nurse	6 years	11 minutes
S4	Female	32	Bachelor	CHSC2	Director of Public Health	4 years	15 minutes
S5	Female	50	Bachelor	CHSC3	Head nurse	30 years	44 minutes
S6	Female	42	Bachelor	CHSC3	General practitioner	18 years	28 minutes
S7	Male	44	Bachelor	CHSC3	Director	18 years	15 minutes
S8*	Female	35	Bachelor	CHSC3	General practitioner	5 years	110 minutes
S9*	Female	26	Bachelor	CHSC3	General practitioner	4 years	40 minutes
S10*	Female	35	Doctor	CHSC3, Neurology department	Neurologist	6 years	30 minutes
S11	Male	30	Bachelor	CHSC3	Dietitian	8 years	20 minutes
S12*	Female	36	Bachelor	CHSC3	Head nurse	4 years	44 minutes
S13	Male	30	Bachelor	CHSC3	General practitioner	4 years	60 minutes
S14	Female	35	Master	CHSC4	Head nurse	15 years	15 minutes

Note: All participants were interviewed, and * indicated that they were also observed.
