# Supplementary material for: The Challenges in Dementia Three-Level Prevention Services Through Integrated Care Theory: Insights from an Ethnographic Study
Source: Int J Integr Care. 2026 Aug 10;26(3):8. doi: 10.5334/ijic.9106 (PMC13475528; doi:10.5334/ijic.9106)
Supplement: Supplementary file 6 [file ijic-26-3-9106-s6.pdf]

Supplementary File 6. Characteristics of service providers from Dementia specialist clinics (n=6)

Code	Gender	Age	Education	Institution	Position	Dementia work experience	Interview time
Y1*	Female	44	PhD	DSC1	Neurologist	15 years	30 minutes
Y2	Male	32	PhD	DSC2	Neurologist	7 years	NA
Y3	Female	26	Master Candidate	DSC1	Neurology Resident	2 years	NA
Y4	Female	25	Master Candidate	DSC1	Neurology Resident	2 years	NA
Y5*	Female	25	PhD Candidate	DSC2	Volunteer, researcher	2 years	90 minutes
Y6	Female	35	PhD	DSC1	Nurse, researcher	7 years	60 minutes

Note: * indicated that they were also interviewed; NA, not available.
