# Supplementary material for: The Challenges in Dementia Three-Level Prevention Services Through Integrated Care Theory: Insights from an Ethnographic Study
Source: Int J Integr Care. 2026 Aug 10;26(3):8. doi: 10.5334/ijic.9106 (PMC13475528; doi:10.5334/ijic.9106)
Supplement: Supplementary file 7 [file ijic-26-3-9106-s7.pdf]

Supplementary File 7. Characteristics of older adults and their family members from Dementia specialist clinics (n=16)

Older adults					Family members				
Code	Gender	Age	Diagnosis	Relationships with older adults	Code	Age	Marital status	Education	Occupation
L1	Female	63	MCI	Daughter	J1*	35	Married	University	Designer
L2	Female	86	AD, Depression, Sleep disorders	Daughter	J2*	54	Married	Polytechnic school	Nurse
L3	Female	60	Mixed type dementia	Daughter	J3*	36	Married	University	Civil servant
L4	Male	88	Brain atrophy, MCI	Daughter	J4*	58	Married	Junior college	Teacher
L5	Male	83	MCI, Cerebral ischemia	Daughter	J5*	55	Married	University	Laborer
L6	Female	62	AD	Daughter	J6*	35	Married	University	Laborer
L7	Male	85	Cerebral infarction, Hypertension, AD	Wife	J7*	82	Married	polytechnic school	Teacher
L8	Female	68	AD	Husband	J8*	69	Married	Junior college	Enterprise Managemen t

Note: MCI, Mild cognitive impairment; AD, Alzheimer disease; * indicated that they were also interviewed.
