# Supplementary material for: The Challenges in Dementia Three-Level Prevention Services Through Integrated Care Theory: Insights from an Ethnographic Study
Source: Int J Integr Care. 2026 Aug 10;26(3):8. doi: 10.5334/ijic.9106 (PMC13475528; doi:10.5334/ijic.9106)
Supplement: Supplementary file 8 [file ijic-26-3-9106-s8.pdf]

Supplementary File 8. The programs involved in dementia three-level prevention services

Services	The description of services
**Dementia Screening Program**	● **Service bodies**: It was a temporary program, launched by the Disease Control Department, led by a psychiatric hospital, and implemented by community health service centers.<br>● **Organizations involved**: A psychiatric hospital, community health service centers, and neighbourhood committees.<br>● **Goals and values**: It aimed to deliver dementia-related services in communities, such as health education, screening, referral, and diagnosis for individuals with suspected mild cognitive impairment or dementia.<br>● **Cooperation mechanism**: Hierarchical governance.<br>● **Professionals involved**: They usually included specialists, general practitioners, nurses, and other medical technicians. The specialists were from the psychiatric hospital, and the general practitioners, nurses, and other medical technicians were from community health service centers.<br>● **Professionals function**: General practitioners, nurses, and other medical technicians were responsible for conducting dementia screening, while specialists were responsible for supervising the screening and diagnosing suspected patients.<br>● **Benefits-sharing mechanism**: The Disease Control Department launched the temporary project and provided financial support to the psychiatric hospital. The psychiatric hospital provided screening, diagnosis, treatment, and other support, while community health service centers were required by higher authorities, namely the Disease Control Department and psychiatric hospital, to conduct screening and fulfill their responsibilities.

Services	The description of services
**Dementia-Friendly Communities**	● **Service bodies**: These projects were usually issued by local Civil Affair department, applied and implemented by public welfare organizations or community's comprehensive older adults service centers.<br>● **Organizations involved**: Public welfare organizations or community's comprehensive older adults service centers, volunteer service organizations, research teams, and the third-party technology companies.<br>● **Goals and values:** They were aimed at providing dementia health education, screening, non-pharmacological interventions, care support, etc., for community residents.<br>● **Cooperation mechanism**: A verbal or written agreement between public welfare organizations or community's comprehensive older adults service centers and the research teams.<br>● **Professionals involved**: They typically comprised social workers, volunteers, researchers, and third-party technology company staff. Social workers were typically employed by public welfare organizations or community's older adults service centers. The volunteers (usually college students, unemployed residents, etc.) were recruited by volunteer service organizations or research teams. In some cases, volunteers also served as researchers.<br>● **Professionals function**: Social workers were responsible for designing, coordinating, and delivering dementia health education and screening services. Research teams were responsible for ensuring scientific rigor, and providing training and supervision for health education and screening services. Social volunteers were responsible for implementing screening. The third-party technology company staff were responsible for developing the screening apps and/or websites.<br>● **Benefits-sharing mechanism**: The Civil Affairs Department launched projects on a regular basis and provided financial support to the implementing organizations.<br>● Public welfare organizations or community's older adults service centers aimed to enhance their reputation and expand their service offering. Meanwhile, research teams sought to explore new avenues of study and gather valuable data. Volunteer organizations were driven by their mission to foster a culture of volunteerism and fulfill their social mandate.

Services	The description of services
**Medical Consortia**	● **Service bodies**: The projects were usually issued by Health Commission, and implemented by tertiary hospitals and community health service centers.<br>● **Organizations involved**: The main implementing bodies were usually tertiary hospitals and community health service centers.<br>● **Goals and values:** They were aimed at promoting patient diversion and enhancing service capabilities of community health service centers.<br>● **Cooperation mechanism**: The collaboration within Medical Consortia followed a hierarchical governance model, typically with tertiary hospitals leading and supporting community health service centers in the delivery of clinical services such as diagnosis and treatment.<br>● **Professionals involved**: The teams usually comprised specialists, general practitioners, nurses, and other health professionals. Typically, the specialists and dementia-specialist nurses were from tertiary hospitals, while the general practitioners, community nurses, and other staff came from community health service centers.<br>● **Professionals function**: Ideally, general practitioners conducted initial screenings and provided empirical treatment for community residents suspected of mild cognitive impairment or dementia, and transferred them to tertiary hospitals. Specialists at tertiary hospitals then performed definitive diagnoses, prescribed treatment, and developed personalized care plans in collaboration with dementia-specialist nurses. Finally, general practitioners and community nurses were responsible for the follow-up and the management with guidance and supervision from the specialist team.<br>● **Benefits-sharing mechanism**: The projects were mandated by Health Commission, with the goal of promoting public health through collaboration between tertiary hospitals and community health service centers. As part of this, tertiary hospitals fulfilled their public service mandate by providing technical support to community health service centers.
**Long-term care**	● **Service bodies**: Long-term care services operated as a multi-party co-construction system, which led by government,

Services	The description of services
**services**	participated by society, and operated by market.<br>● **Organizations involved**: For residents living in communities, the implementing bodies were usually long-term care service companies, and families.<br>● **Goals and values:** The system aimed to provide accessible, affordable, and quality care to older adults with functional impairments, thereby enhancing their quality of life and reducing family burden.<br>● **Cooperation mechanism**: A networked cooperation model was common. The government purchased services from qualified long-term care service companies. Communities, hospitals, community health service centers, and long-term care service companies formed networks to promote the services and enable referrals.<br>● **Professionals involved**: The care teams were typically composed of trained assessors, nurse aides, managers and family caregivers.<br>● **Professionals function**: Trained assessors conducted comprehensive and standardized needs assessments to determine eligibility, classify care levels, and formulate the initial individualized care plan. Managers acted as the coordinators to translate the assessment plan into actionable services,including matching clients with suitable nurse aides, coordinating schedules, and monitoring service quality. Nurse aides delivered care services, centered on assisting with Activities of Daily Living, such as personal hygiene, dressing, feeding, and ambulation. Family caregivers provided continuous emotional support, living environment management, and supplemental care. Their critical role was to sustain care routines outside formal service hours, observe and report changes, and collaborate closely with managers and aides.<br>● **Benefits-sharing mechanism**: This mechanism operated not as a simple fee-for-service model, but as a value-based ecosystem. Financial resources flowed through the payer to the provider organization, which deployed managers to orchestrate care delivered by aides, all based on the assessor's initial blueprint. Benefits was created through better health outcomes, efficiency, and satisfaction, and was shared among all participants.
