# Supplementary material for: The Challenges in Dementia Three-Level Prevention Services Through Integrated Care Theory: Insights from an Ethnographic Study
Source: Int J Integr Care. 2026 Aug 10;26(3):8. doi: 10.5334/ijic.9106 (PMC13475528; doi:10.5334/ijic.9106)
Supplement: Supplementary file 9 [file ijic-26-3-9106-s9.pdf]

Supplementary File 9. The identified challenges through rainbow model of integrated care

**Challenges**	**Dementia three-level prevention services**				
	Dementia Program	Screening	DFCs	Medical Consortia	Long-term care services
**Challenges from system integration**					
Challenge 1: Lack of formal task plans from administrative organization			√		
Challenge 2: Dementia not involved in health management				√	
Challenge 3: Ineffective implementation of family physician system				√	
Challenge 4: Uncharged non-pharmacological interventions				√	
**Challenges from organizational integration**					
Challenge 1: Unformed atmosphere of collaboration	√		√		
Challenge 2: Inadequate engagement of neighborhood committees			√		
Challenge 3: Verbal agreements			√		
Challenge 4: Inappropriate values			√		
Challenge 5: Residents' distrust			√	√	
Challenge 6: Lack of referral			√	√	
Challenge 7: Absence of common goals and benefits-sharing mechanisms				√	
Challenge 8: Inadequate resource sharing				√	
Challenge 9: Uncoordinated functional positions				√	
Challenge 10: Unformed cross-institutional dementia management team				√	
**Challenges from professional integration**					
Challenge 1: Limited human resources	√		√		
Challenge 2: Underutilization of professional expertise			√		
Challenge 3: Shortage of volunteers			√		
Challenge 4: Lack of motivation				√	
Challenge 5: Underutilized potential for healthcare professionals				√	
Challenge 6: Insufficient Capability				√	
Challenge 7: Incomplete team structure				√	
**Challenges from clinical integration**					
Challenge 1: Difficulty in screening	√		√	√	
Challenge 2: Unavailable non-pharmacological interventions				√	

Challenge 3: Lack of comprehensive assessment, goals, and care plans			√	
Challenge 4: Delays in accessing long-term care services				√
Challenge 5: Unmet personalized long-term care needs				√
Challenge 6: Standardized process for long-term care				√
Challenge 7: Low level of active participation of residents and their families	√	√	√	
**Additional Challenges**				
Challenge 1: Lack of dementia-related knowledge	√	√	√	
Challenge 2: Stigma	√	√	√	
Challenge 3: Difficulty in caring work				√
Challenge 4: Lack of professional and convenient day care institutions				√

Note: DFCs, Dementia friendly communities; CHSCs, Community health service centers; DSCs, Dementia special clinics.
